# Our Agents

**Published:** 1885-12

**Authors:** 


OUR AGENTS.—Mr. W. R. Case, of Philadelphia—William Wood & Co.'s and F. A. Davis' traveling agent—and Messrs. Dixon & Neatherry, the well known publishers of *The Poets and Poetry of Texas* (by Mr. Dixon) are authorized to solicit, receive and receipt for subscriptions to this JOURNAL, and also applications for membership in the P. M. B. A. of Texas. Messrs. Dixon & Neatherry have contracted with us for the publication of the *Biography of Cotemporary Physicians of Texas*, and will make a personal canvass of the State in the interest of that work, now in preparation. Mr. Case is their agent, authorized to solicit subscriptions and to make all necessary arrangements with physicians for the insertion of their biographies. We commend these gentlemen to the profession as worthy in every way of their confidence.

